# AOPM: Application of Antioxidant Protein Classification Model in Predicting the Composition of Antioxidant Drugs

**DOI:** 10.3389/fphar.2021.818115

**Published:** 2022-01-18

**Authors:** Yixiao Zhai, Jingyu Zhang, Tianjiao Zhang, Yue Gong, Zixiao Zhang, Dandan Zhang, Yuming Zhao

**Affiliations:** ^1^ College of Information and Computer Engineering, Northeast Forestry University, Harbin, China; ^2^ Department of Neurology, the Fourth Affiliated Hospital of Harbin Medical University, Harbin, China; ^3^ Department of Obstetrics and Gynecology, the First Affiliated Hospital of Harbin Medical University, Harbin, China

**Keywords:** antioxidant proteins, random forest, MRMD, antioxidant drugs, drug screening and discovery

## Abstract

Antioxidant proteins can not only balance the oxidative stress in the body, but are also an important component of antioxidant drugs. Accurate identification of antioxidant proteins is essential to help humans fight diseases and develop new drugs. In this paper, we developed a friendly method AOPM to identify antioxidant proteins. 188D and the Composition of k-spaced Amino Acid Pairs were adopted as the feature extraction method. In addition, the Max-Relevance-Max-Distance algorithm (MRMD) and random forest were the feature selection and classifier, respectively. We used 5-folds cross-validation and independent test dataset to evaluate our model. On the test dataset, AOPM presented a higher performance compared with the state-of-the-art methods. The sensitivity, specificity, accuracy, Matthew’s Correlation Coefficient and an Area Under the Curve reached 87.3, 94.2, 92.0%, 0.815 and 0.972, respectively. In addition, AOPM still has excellent performance in predicting the catalytic enzymes of antioxidant drugs. This work proved the feasibility of virtual drug screening based on sequence information and provided new ideas and solutions for drug development.

## Introduction

In the process of biological metabolism, reactive oxygen species (ROS) are produced. The antioxidant system in the organism can eliminate ROS, but there is a limit. Too high concentrations of ROS are not eliminated in time and will cause oxidative stress (OS) (Birben et al., 2012; Yang et al., 2020; Zhao S et al., 2021). According to research, OS response plays an important role in the pathogenesis of many diseases. Long-term response to OS will destroy the structure of macromolecules and even affect the senescence and death of cells. Research by Azhwar Raghunath’s team (Raghunath et al., 2018) has shown that the protective effect against oxidative stress is a *cis*-acting element of antioxidant proteins in the regulation of Nrf2 target genes, which plays a key role in redox homeostasis. Therefore, antioxidant proteins have been used in the development and screening of antioxidant drugs, which can treat cancer, neurodegenerative diseases, cardiovascular, metabolic and other diseases with oxidative stress (Liguori et al., 2018; Eleutherio et al., 2021; Zia et al., 2021).

Traditional antioxidant drug screening and discovery are carried out through biochemical experiments, which not only has a long time period and high cost, but also has the risk of failure in experiments (Lv et al., 2020a; Cheng et al., 2020; Cheng Y et al., 2021; Lv Z et al., 2021; Dong et al., 2021; Goto et al., 2021; Zeng et al., 2022). With the continuous improvement of computer technology and genome databases, methods such as data mining and machine learning are more and more widely used in biological information, drug screening and other fields (Cheng et al., 2018; Wang et al., 2018; Ding et al., 2019; Wang et al., 2019; Zeng et al., 2020a; Zhang CH et al., 2020; Zhang J et al., 2020; Lyu et al., 2020; Zhao X et al., 2021; Niu et al., 2021). In recent years, many researchers have been exploring machine learning models suitable for identifying antioxidant proteins. The Feng team adopted the Naive Bayesian method and the AodPred model to identify antioxidant proteins, which proposed in 2013 (Feng et al., 2013) and 2016 (Feng et al., 2016) respectively. AodPred is based on a vector machine model with 3 spaced residual pairs, which is significantly better than Naive Bayes but its ability to identify antioxidant proteins is still limited. In 2016, the integration method used by Zhang showed that the secondary structure of proteins helps distinguish antioxidant proteins from non-antioxidant proteins, but the method of feature extraction for this model is complicated and time-consuming. Subsequently, both Xu et al. (2018), Meng et al. (2019) adopted the support vector machine model to identify the target protein. In 2020 (Zhai et al., 2020), our team explored the random forest combined with SMOTE to identify antioxidant proteins. The ability to identify antioxidant proteins has improved a lot compared to the original Feng. However, when dividing the training set and the data set for the three of them, there are ambiguities and the test set does not reflect the original data distribution. In addition, these researchers did not consider whether the model can be applied to the screening of antioxidant drugs and other practical problems when they created the model for identifying antioxidant proteins (Wang et al., 2020; Chen et al., 2021). In fact, this is a very good idea, but no one has done so yet.

**FIGURE 1:**
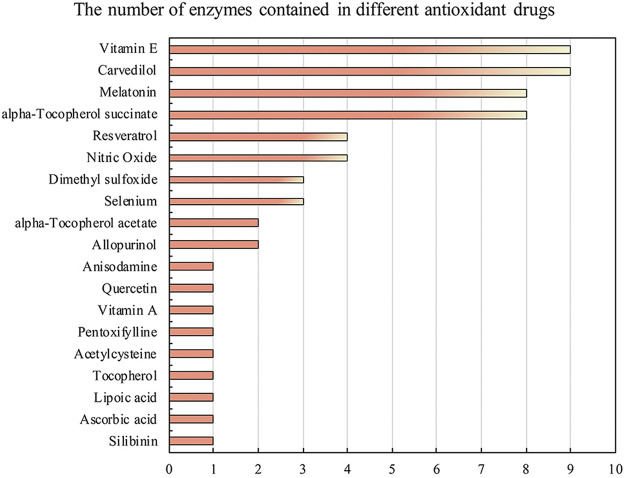
The function structure of AOPM.

In response to these problems, we exploited a method, AOPM, which is a pipeline for identifying antioxidant protein sequence data. This model can also be used in the application of virtual antioxidant drug screening. To facilitate understanding, Figure 1 shows the flow chart of AOPM. The feature extraction part adopted amino acid composition and physical and chemical properties to extract 188-dimensional features (Liu T et al., 2020) from protein sequences, which was same with Xu. The Composition of k-spaced Amino Acid Pairs (CKSAAP) (Usman and Lee, 2019) was also adopted as the feature extraction methods. In addition, we preferred a very mature feature selection method, the Max-Relevance-Max-Distance algorithm (MRMD) (Zou et al., 2016; Lv et al., 2020b), which was based on the Pearson correlation coefficient and could be exploited to single out the best feature subset for reducing the computational complexity and noise. On the contrary, we chose a 5-fold cross-validation as the model selection method and random forest (Liaw and Wiener, 2002; Lv et al., 2019) as the classifier, which has the characteristics of a fast running speed and less overfitting, rather than the very popular support vector machine.

Finally, on the antioxidant protein test dataset, after AOPM processing. The sensitivity (SN), specificity (SP), accuracy (ACC), Matthew’s Correlation Coefficient (MCC) and an Area Under the Curve (AUC) reached 87.3, 94.2, 92.0%, 0.815 and 0.972, respectively, which were significantly better than the results with the AodPred and Zhai. In addition, AOPM still has excellent performance in identifying the proteins that make up antioxidant drugs, providing new ideas for exploring the research of drug components. In addition, when using AOPM to predict the 36 protein sequences located in the DrugBank (Wishart et al., 2018) data set, 11 of them were judged to have the function of antioxidants. Among them, Superoxide dismutase [Cu-Zn] is indeed a protein with antioxidant capacity (Dzięgielewska-Gęsiak et al., 2014; Tiwari et al., 2019; Ściskalska et al., 2020). This work proved the feasibility of virtual drug screening based on sequence information and provided new ideas and solutions for drug development (Liu J et al., 2020; Jakhar et al., 2020; Shaker et al., 2021; Yan et al., 2021; Zhu et al., 2021).

## Materials and Methods

### Availability of Data and Materials

We first collected proteins with antioxidant activities from the antioxidant protein database (AOD) (Feng et al., 2017). AOD (Antioxidant Protein Database) is a manually planned and experimentally verified antioxidant protein database. The data and information are extracted from UniProtKB/Swiss-Prot (release 2016_11) according to the following steps: 1) only proteins with experimentally proven antioxidant activities were selected; and 2) ambiguous proteins were excluded, such as those containing nonstandard letters like “B,” “X,” and “Z”. After this rigorous screening, we obtained 710 protein sequences as the original positive samples for the experiment. The negative samples were 1552 PDB proteins with identical values <20%, which were picked by PISCES-culled.

Then we divided the original data set into training set and test set according to the ratio of 4:1. The training set contains 568 antioxidant proteins and 1242 non-antioxidant proteins. The rest of the data are the test set, including 142 antioxidant proteins and 310 non-antioxidant proteins. The detailed data set information is shown in Table 1.

**TABLE 1:** Antioxidant protein datasets information.

Dataset	Sample	Class	Positive num	Negative num
Train dataset	1810	2	568	1242
Test dataset	452	2	142	310

**FIGURE 2:**
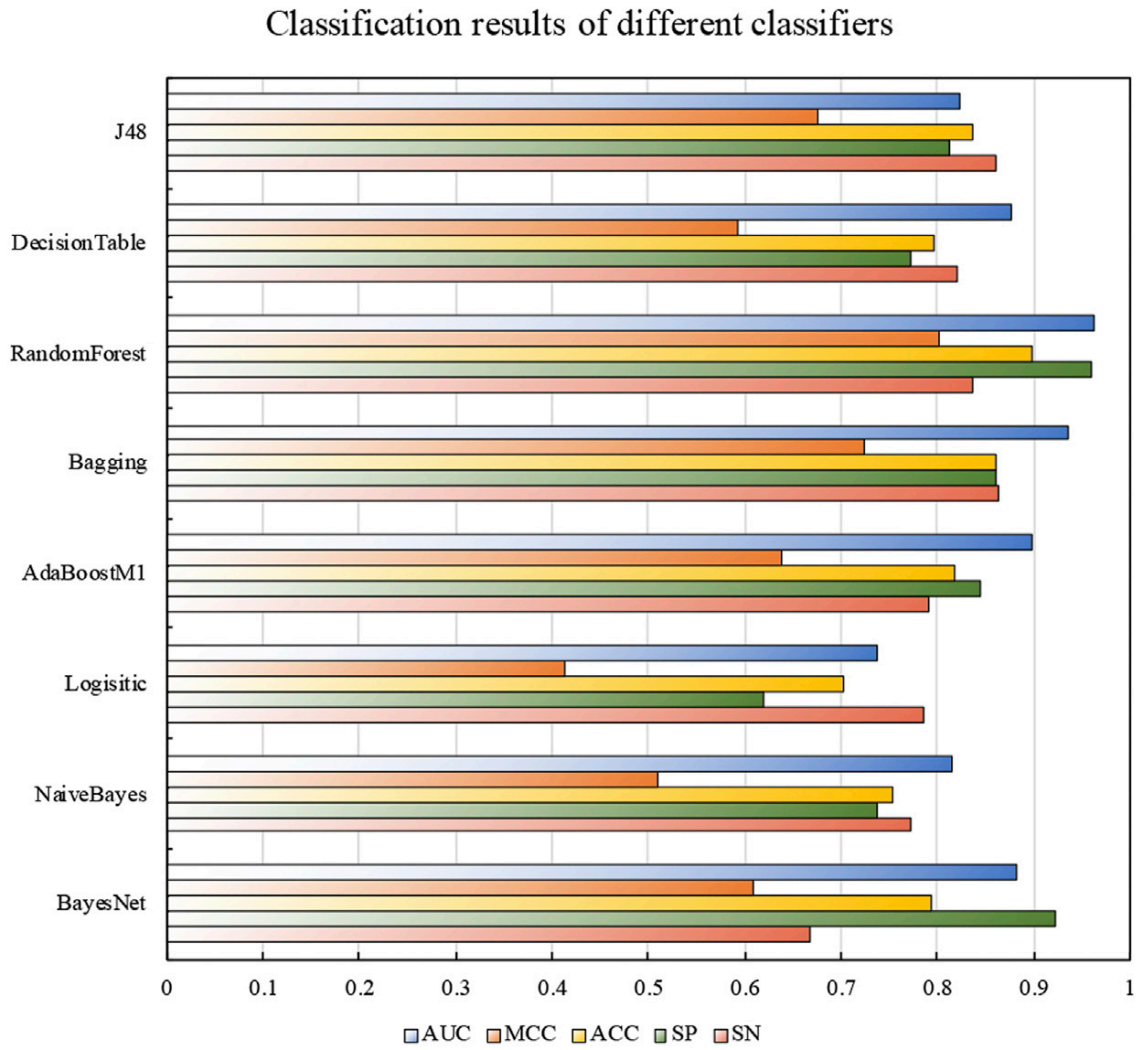
The number of catalytic enzymes contained in different antioxidants. The catalytic enzymes of antioxidant drugs are diverse. For example, for antioxidant drugs such as Vitamin E and Carvedilol, the number of enzymes that can catalyze is as high as 9 types. Of course, some antioxidant drugs can only be catalyzed by a specific enzyme, Such as Anisodamine, Silibinin, and Lipoic acid.

In addition, in the DrugBank database, 19 drugs were found to have antioxidant properties. On this basis, we screened out 36 protein sequences of enzymes that play a catalytic role in antioxidant drugs. This data set was used to test the prediction performance of AOPM in the real data set. The UniProt IDs of 36 protein sequences were shown in Table 2. In addition, a protein can act as a catalytic enzyme in different antioxidants, as shown in Figure 2.

**TABLE 2:** The UniProt ID of 36 protein sequences.

UniProt ID	Drug	Type
P47989	Carvedilol, Allopurinol	enzyme
P16662	Carvedilol	enzyme
P06133	Carvedilol	enzyme
P22309	Carvedilol, Silibinin	enzyme
Q16881	Ascorbic acid, Selenium	enzyme
P00441	Vitamin E, alpha-Tocopherol succinate	enzyme
Q96I15	Selenium	enzyme
P16435	Lipoic acid	enzyme
P15559	Vitamin E, alpha-Tocopherol succinate	enzyme
P05164	Melatonin	enzyme
P78329	Tocopherol, alpha-Tocopherol acetate	enzyme
P14902	Melatonin	enzyme
P09601	Vitamin E, alpha-Tocopherol succinate	enzyme
P46597	Melatonin	enzyme
P05091	Nitric Oxide	enzyme
Q06278	Allopurinol	enzyme
Q03154	Acetylcysteine	enzyme
P11511	Melatonin	enzyme
P04798	Melatonin, Resveratrol, Carvedilol	enzyme
P05177	Nitric Oxide, Pentoxifylline, Melatonin, Resveratrol, Carvedilol	enzyme
Q16678	Melatonin, Resveratrol	enzyme
O43174	Vitamin A	enzyme
P20813	Nitric Oxide	enzyme
P33261	Melatonin, Dimethyl sulfoxide	enzyme
P10632	Quercetin	enzyme
P11712	Melatonin, Carvedilol	enzyme
P10635	Dimethyl sulfoxide, Anisodamine, Carvedilol	enzyme
P05181	Carvedilol	enzyme
P08684	Vitamin E, Nitric Oxide, Dimethyl sulfoxide, Resveratrol, Tocopherol, alpha-Tocopherol acetate, Carvedilol	enzyme
P48506	Vitamin E, alpha-Tocopherol succinate	enzyme
P00390	Selenium	enzyme
P09210	Vitamin E, alpha-Tocopherol succinate	enzyme
P21266	Vitamin E, alpha-Tocopherol succinate	enzyme
P78417	Vitamin E, alpha-Tocopherol succinate	enzyme
P09211	Vitamin E, alpha-Tocopherol succinate	enzyme

### Under Sampling Processing

The number of antioxidant proteins is relatively small. Although the ratio of the number of antioxidant proteins to the number of non-antioxidant proteins in the training set is 1:2, in order to find the characteristics of more clearly distinguishing antioxidant proteins, we performed the training dataset under sampling process. In this study, we selected five different under sampling methods in KEEL (Triguero et al., 2017) to resemble the sample. These five methods included CNN_TomekLinks, CPM, NCL, OSS, and RandomUnderSample.

In the processed data set, the number of antioxidant proteins and non-antioxidant proteins are not exactly the same. The operation of this step is to highlight the characteristics that are beneficial to distinguish antioxidant proteins as much as possible.

### Feature Extraction

In order to obtain sequence information more comprehensive, we adopted feature extraction methods from two perspectives, including sequence composition and the physical and chesmical properties of amino acids (Zulfiqar et al., 2021a; Cheng L et al., 2021; Zhang et al., 2021). Among them, we used the 188D method to extract the characteristic information about the physical and chemical properties of the sequence, and select the Composition of k-spaced Amino Acid Pairs (CKSAAP) (Chen et al., 2007) method to obtain the characteristic information about the sequence composition (Naseer et al., 2020; Long et al., 2020).

#### 188D

The expression form of the amino acid sequence is a string sequence or a discrete multidimensional vector. The multidimensional vector representation method lacks the content of amino acid position information and arrangement sequence; therefore, the research value is small. The descriptive form of the string sequence is that each of the 20 amino acids is represented by a letter, and the letter sequence is used to represent a protein sequence. Since the classifier cannot recognize the string, the feature extraction function of this project uses the 188D feature extraction method to extract useful numerical information from the amino acid sequence as the input of the model.

The 188D feature extraction method is based on 188 features extracted based on protein sequence information and physical and chemical properties. In 2003, the researchers proposed this feature extraction method, which combines the physical and chemical properties of proteins. The 188-dimensional features can be divided into two categories: one is composed of 20 amino acids, and the other is physical and chemical properties, including hydrophobicity, polarity, normalized van der Waals volume, surface tension, charge, polarizability, solvent accessibility, and secondary structure. The dimensions of the different characteristics are shown in Table 3.

**TABLE 3:** Ingredients contained in the 188-dimensional feature of a protein.

Physicochemical property	Dimensions
Amino acid composition	20
Hydrophobicity	21
Van der Waals volume	21
Polarity	21
Polarizability	21
Charge	21
Surface tension	21
Secondary structure	21
Solvent accessibility	21
Total	188

First, we calculated the corresponding frequencies of the 20 amino acids, which can be expressed as $n_{1,}n_{2,}n_{3,}\cdots n_{20}$ , where L is the length of the sequence and $F_{ai}$ is the frequency of the i -th amino acid. The frequency formula for the appearance of 20-dimensional amino acids is as follows.

$$\begin{array}{c} F_{ai}=\frac{n_{i}}{L},\;i\in \left[1,20\right] \end{array}$$ (1)

The 20 amino acids are divided into three types according to their physical and chemical properties. These three categories include content (C), distribution (D) and bivalent frequency (B), which are adopted to describe the physical and chemical properties of proteins. Table 4 shows the amino acid grouping table of the 8 physicochemical properties.

**TABLE 4:** List of the 3 categories divided according to the physical and chemical properties of proteins.

Physicochemical property	Ⅰ	Ⅱ	Ⅲ
Hydrophobicity	RKEDQN	GASTPHY	CVLIMFW
Van der Waals volume	GASCTPD	NVEQIL	MHKFRYW
Polarity	LIFWCMVY	PATGS	HQRKNED
Polarizability	GASDT	CPNVEQIL	KMHFRYW
Charge	KR	ANCQGHILMFPSTWYV	DE
Surface tension	GQDNAHR	KTSEC	ILMFPWYV
Secondary structure	EALMQKRH	VIYCWFT	GNPSD
Solvent accessibility	ALFCGIVW	RKQEND	MPSTHY

First, we calculated the frequency characteristics of the three categories, which are represented as $CS_{1}$ , $CS_{2}$ , and $CS_{3}$ . Their frequency characteristics are expressed as:

$$\begin{array}{c} \left(F_{q1},F_{q2},F_{q3}\right)=\left(\frac{CS_{1}}{L},\frac{CS_{2}}{L},\frac{CS_{3}}{L}\right) \end{array}$$ (2)

For each group, the first and 25, 50, 75 and 100% dipeptide chain positions are represented by $DS_{ij}$ , where i is the group number, and the value range is 1–3; j is the dipeptide chain position, and the value range is 1–5.

$$\begin{array}{c} \left(F_{q4},F_{q5},F_{q6},F_{q7},F_{q8}\right)=\left(\frac{DS_{11}}{L},\frac{DS_{12}}{L},\frac{DS_{13}}{L},\frac{DS_{14}}{L},\frac{DS_{15}}{L}\right) \end{array}$$ (3)

$$\begin{array}{c} \left(F_{q9},F_{q10},F_{q11},F_{q12},F_{q13}\right)=\left(\frac{DS_{21}}{L},\frac{DS_{22}}{L},\frac{DS_{23}}{L},\frac{DS_{24}}{L},\frac{DS_{25}}{L}\right) \end{array}$$ (4)

$$\begin{array}{c} \left(F_{q14},F_{q15},F_{q16},F_{q17},F_{q18}\right)=\left(\frac{DS_{31}}{L},\frac{DS_{32}}{L},\frac{DS_{33}}{L},\frac{DS_{34}}{L},\frac{DS_{35}}{L}\right) \end{array}$$ (5)

In addition, we also calculated the number of dipeptides from different groups and obtained the parameters $BS_{1}$ , $BS_{2}$ , and $BS_{3}$ so that the frequency of the doublet sequence is calculated as:

$$\begin{array}{c} \left(F_{q19},F_{q20},F_{q21}\right)=\left(\frac{BS_{1}}{L},\frac{BS_{2}}{L},\frac{BS_{3}}{L}\right) \end{array}$$ (6)

In the above formula, $F_{qi}$ represents the i -th feature of a physical and chemical property. A total of (3+3+3×5)=21 feature vectors are extracted from each attribute, and finally, all 21×8=168 feature vectors are extracted from 8 physical and chemical properties. In addition, the 20 amino acid frequencies are added, and finally, 168+20=188 dimensional feature vectors are obtained.

#### Composition of K-Spaced Amino Acid Pairs

The Composition of k-spaced Amino Acid Pairs (CKSAAP) feature delegates the component of amino acids. It represents the frequency calculation of two amino acids separated by k residues. Experiments have confirmed that the three-spaced residue pair feature is beneficial to the classification of antioxidant proteins, so we only adopted k=3 in this method, which selected 400 dimensions. 20 kinds of amino acids were brightly combined in pairs to obtain 400 amino acid pairs. We can calculate the frequency of 400 amino acid pairs in a protein sequence. Then, a 3-spaced feature vector can be defined as:where $n_{ij}$ is the number of times the ij -th amino acid pair appears in a protein sequence and N is the length of the protein sequence. In addition, ij is the amino acid pair of 20 kinds of amino acids in two groups.

$$\begin{array}{c} F_{ij}=\frac{n_{ij}}{N-4} \end{array}$$ (7)

### Feature Selection

Feature selection obtains the most effective feature subset for classification and recognition of the many features (Wang et al., 2010; Mo et al., 2020; Sheng et al., 2021; Wu et al., 2021). That is, it captures a set of “small but precise” classification features with a small probability of error. While reducing the dimensionality of the feature space in this way, it also speeds up the construction of the classifier model (Yu XP et al., 2021; Long et al., 2021; Yang et al., 2021). In AOPM, the Max-Relevance-Max-Distance algorithm (MRMD) was used for feature selection, which was proposed by Zou.

The MRMD score of each feature consists of two parts: the correlation and distance value between the feature and other features. The Pearson correlation coefficient was used to calculate the correlation between features. It represents the degree of linear correlation between features. The larger the absolute value is, the stronger the degree of linear correlation. The value of ${\text{MR}}_{i}$ (max-relevance) for feature i is defined as follows:

$$\max\;MR_{i}=\left|PCC\left(\vec{F_{i}},\vec{C_{i}}\right)\right|$$ (8)

where $\vec{F_{i}}$ is the i -th feature of each instance and $\vec{C_{i}}$ is the i -th target class of each instance. The distance value provides four calculation methods. In addition to the three mature distance calculation methods, there is another method that is, based on the average of the three methods to obtain the distance value. The three traditional methods are Euclidean distance, cosine similarity and Tanimoto coefficient, which are designated by the symbols ${\text{ED}}_{i}$ , ${\text{COS}}_{i}$ , and ${\text{TC}}_{i}$ . The mean of these is designated by the symbol ${\text{MEAN}}_{i}$ . Finally, the value of ${\text{MD}}_{i}$ (max-distance) for feature i is defined as follows:

$$\max\;MD_{i}=ED_{i}$$ (9)

$$\max\;MR_{i}=COS_{i}$$ (10)

$$\max\;MR_{i}={\text{TC}}_{i}$$ (11)

$$\max\;MR_{i}={\text{MEAN}}_{i}$$ (12)

$$\begin{array}{c} {\text{MEAN}}_{i}=\frac{\left({\text{ED}}_{i}+{\text{COS}}_{i}+{\text{TC}}_{i}\right)}{3} \end{array}$$ (13)

According to ${\text{MR}}_{i}$ and ${\text{MD}}_{i}$ , the MRMD score is defined as:

$$\max\left({\text{MR}}_{i}+{\text{MD}}_{i}\right)$$ (14)

All features are arranged in descending order according to the MRMD score. One feature with the highest MRMD score is sequentially added to the feature subset. Then, the feature subset is input into the selected classifier for classification, and the classification accuracies of different feature subsets are recorded. In the end, the feature subset with the highest accuracy and the least number of features is the result of feature selection.

### Random Forest

Random forest is an ensemble algorithm that integrates multiple trees through the idea of ensemble learning. It has been widely used in bioinformatics (Jin et al., 2019; Manavalan et al., 2019a; Manavalan et al., 2019b; Riaz and Li, 2019; Su et al., 2019; Ściskalska et al., 2020; Zeng et al., 2020b; Ao et al., 2021; Zulfiqar et al., 2021b). It consists of N decision trees. After the sample is input into the random forest, each decision tree will get a classification result, and then N trees will get N classification results. Count the voting results of all classification results, and the category with the most votes is the final output.

In our research, we use the random forest as the classifier because it has several advantages that suit our data. The dimensionality of the extracted feature set is high, even after dimensionality reduction, it still belongs to high-dimensional data. Random forest is less affected by parameters, and when processing high-dimensional data, the accuracy is not affected. In addition, using random forest processing, the running speed is fast, there is no need to debug many parameters like SVM, and the time cost is low.

## Results

### Measurement

At present, AOPM can only deal with two classification problems. There are three commonly used evaluation methods, including the independent data set sampling test, the k-fold cross validation and the jack-knife test (Wang et al., 2008; Wei et al., 2014; Wei et al., 2017; Basith et al., 2018; Wei et al., 2018; Lv H et al., 2021; Yu L et al., 2021; Wu and Yu, 2021). To simplify the calculation, we adopted 5-fold cross-validation to compare the classifiers. And test the robustness of the model on the test dataset.

In addition to the commonly used evaluation indicators sensitivity (SN), specificity (SP) and accuracy (ACC), AOPM also provided a Matthew’s Correlation Coefficient (MCC) and an Area Under the Curve (AUC) to evaluate the performance of the ensemble classifier, and the formulas were defined as follows (Liang et al., 2019; Lv et al., 2020c):

$$\begin{array}{c} SN=\frac{TP}{TP+FN} \end{array}$$ (15)

$$\begin{array}{c} SP=\frac{TN}{TN+FP} \end{array}$$ (16)

$$\begin{array}{c} ACC=\frac{TN+TP}{TP+TN+FP+FN} \end{array}$$ (17)

$$\begin{array}{c} MCC=\frac{TP\times TN-FP\times FN}{\sqrt{\left(TP+FN\right)\left(TP+FP\right)\left(TN+FP\right)\left(TN+FN\right)}} \end{array}$$ (18)

where TP is the number of samples judged as positive for the positive class, FP is the number of samples judged as positive for the negative class, FN is the number of samples judged as negative for the positive class, and TN is the number of samples judged as negative for the negative class. MCC is an index used in machine learning to measure the classification performance of two categories. In addition, the AUC value was obtained by calculating the area of the ROC curve and the area surrounded by the *X*- and *Y*-axes, where the *X*- and *Y*-axes of the ROC curve were (1-SP) and SN, respectively.

### The Influence of Different Combinations of Feature Selection Methods on the Final Result

According to existing research, a series of feature extraction methods have been proved to be effective for the classification of antioxidant proteins, such as g-gap dipeptide feature, CTD, 188D, etc. However, the existing methods all use a certain method alone, and do not use them in combination. Therefore, in the planning stage of the experiment, we chose CKSAAP and 188D to find the most suitable combination of features for the target protein. Among them, CKSAAP is divided into pairs containing g-spacer residues (g = 0, 1, 2, 3, 4, 5). The experimental results of the random forest classifier and 5-fold cross-validation on the training set were shown in Table 5.

**TABLE 5:** Classification results of different under-sampling methods on the train dataset.

Feature extration methods	Performance metrics (%)				
	SN	SP	ACC	MCC	AUC
188D	**0.877**	0.917	0.897	0.795	0.964
188D + CKSAAP (g = 0)	0.840	0.945	0.893	0.79	0.961
188D + CKSAAP (g = 1)	0.849	0.942	0.895	0.794	0.960
188D + CKSAAP (g = 2)	0.833	0.947	0.890	0.785	0.961
188D + CKSAAP (g = 3)	0.836	**0.960**	**0.898**	**0.802**	**0.964**
188D + CKSAAP (g = 4)	0.833	0.945	0.889	0.783	0.961
188D + CKSAAP (g = 5)	0.827	0.942	0.885	0.774	0.959

Bold values indicates the highest value of each indicator.

Only 188D is selected as the feature extraction method, and the SN value reaches 0.877, which was the highest value of all the combination methods, but other indicators are not ideal. When g = 3 for CKSAAP and 188D, all the values except SN are excellent. The SP, ACC, MCC, and AUC were 0.960, 0.898, 0.802, and 0.964, respectively.

### The Impact of the Training Data Set Random Under Sampling of the Results

In order to compare the most suitable under-sampling methods for the antioxidant protein data set, we chose four under-sampling methods, including CNN_TomekLinks, CPM, OSS, and RandomUnderSample, to process the training data separately. At the same time, we followed the single-variable principle. All the parameters in the feature extraction and feature selection of the five sets of data were exactly the same. Finally, 5-fold cross-validation was adopted to obtain the classification effect of the model in the random forest classifier. The classification effect of 5 sets of data is shown in Table 6.

**TABLE 6:** Classification results of different under-sampling methods on the train dataset.

Under-sampling methods	Performance metrics (%)				
	SN	SP	ACC	MCC	AUC
OSS	0.919	0.802	0.872	0.732	0.951
CNNTomekLink	**0.952**	0.631	0.868	0.641	0.892
CPM	0.944	0.576	0.836	0.584	0.892
RUS	0.836	0.960	0.898	**0.802**	**0.964**
Without under-sampling	0.79	**0.966**	**0.911**	0.79	0.96

Bold values indicates the highest value of each indicator.

After the random under-sampling method was used, the MCC and AUC of the model reach 0.802 and 0.964, which were higher than those obtained by other under-sampling methods and direct classification. In addition, SP and ACC have the highest value among all under sampling methods.

### Comparison With Other Traditional Classifiers

**FIGURE 3:**
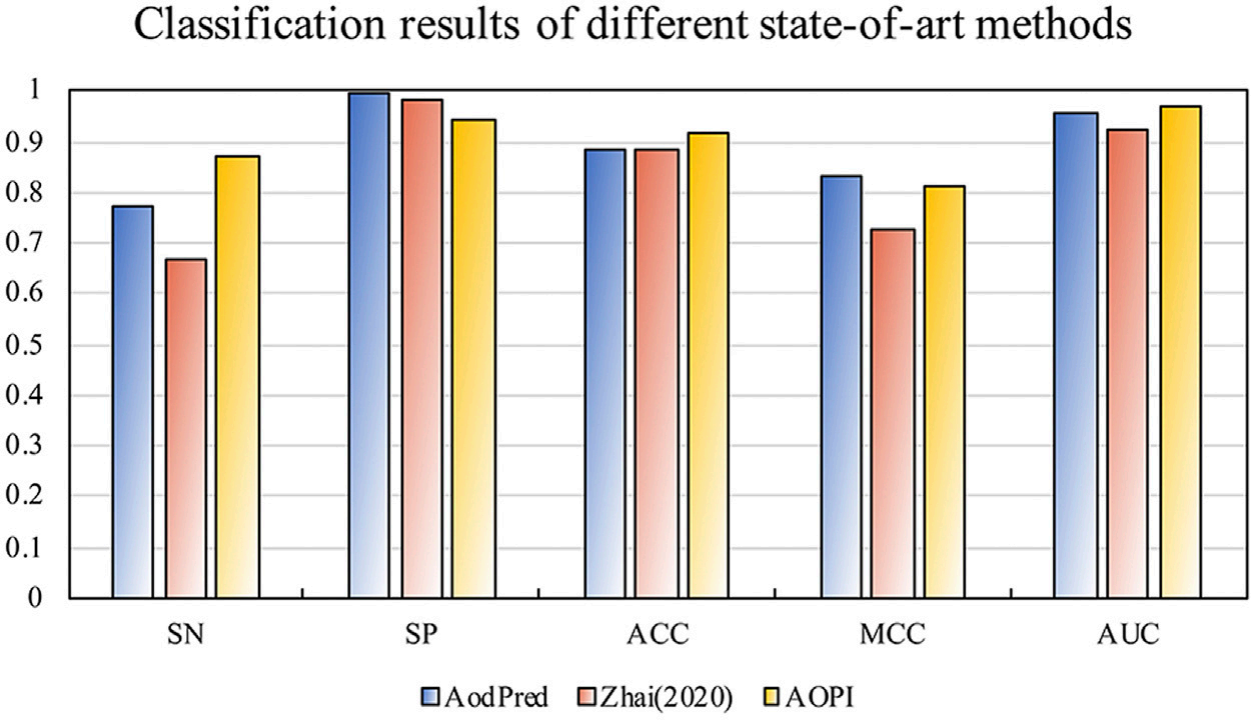
The classification results of different classifiers on the train dataset. The SP, ACC, MCC, and AUC of random forest were much higher than other traditional classifiers, which were 0.960, 0.898, 0.802, and 0.964, respectively. Compared with the Bagging classifier with the highest SN value, the SN value reaches 0.836, which was nearly lower than the highest value of 0.027.

In order to find the most suitable classifier, we selected 8 traditional machine learning classifiers for comparison: BayesNet, naive Bayes, logistic function, AdaBoostM1, bagging, random forest, decision table, and J48. In addition, the results obtained after random under sampling and MRMD processing of the training data set was measured by 5-fold cross-validation in different classifiers. Figure 3 shows the classification results of the training dataset on different classifiers.

Compared with most basic classifiers, random forest showed an exciting classification effect, all indicators were very competitive in all classifiers. It was obviously that the SP, ACC, MCC, and AUC of random forest were much higher than other traditional classifiers, which were 0.960, 0.898, 0.802, and 0.964, respectively. Compared with the Bagging classifier with the highest SN value, the SN value reaches 0.836, which was nearly lower than the highest value of 0.027.

### Comparison With the State-of-the-Art Methods

**FIGURE 4:**
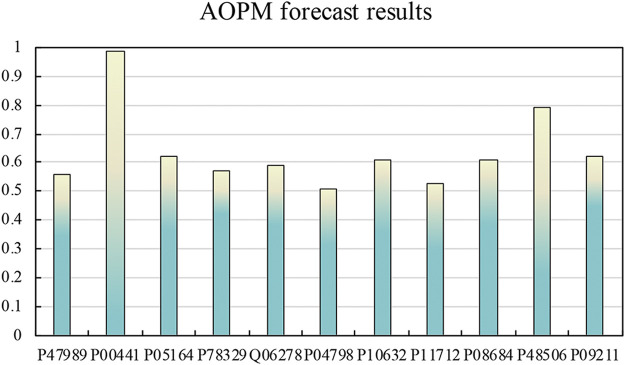
The classification results of the state-of-art methods on the test dataset. The SN, ACC, and AUC of AOPM were 0.873, 0.920, and 0.972, respectively. That was much higher than that of AodPred and Zhai, whose SN was higher than 0.99 and 0.204, respectively.

In order to verify the robustness of AOPM, we chose to compare with two existing methods. They are the AodPred developed by the Feng team and the random forest model developed by ourselves in 2020. Because our data set is different from the existing method, we retrained the model according to the corresponding method and applied it on the same test dataset to get the following results. Figure 4 shows the classification results of the test dataset on the state-of-the-art methods.

It was obviously that the SN, ACC, and AUC of AOPM were much higher than that of AodPred and Zhai, whose SN was higher than 0.99 and 0.204, respectively, indicating that AOPM was more sensitive to the classification of target proteins, which was also consistent with our goal. Although the SP value was slightly lower than the two first, MCC value was higher than Zhai and tinier lower than AodPred, this did not prevent AOPM from being a model with excellent classification effects.

### Predicted Results of Protein Contained in Antioxidant Drugs

**FIGURE 5:**
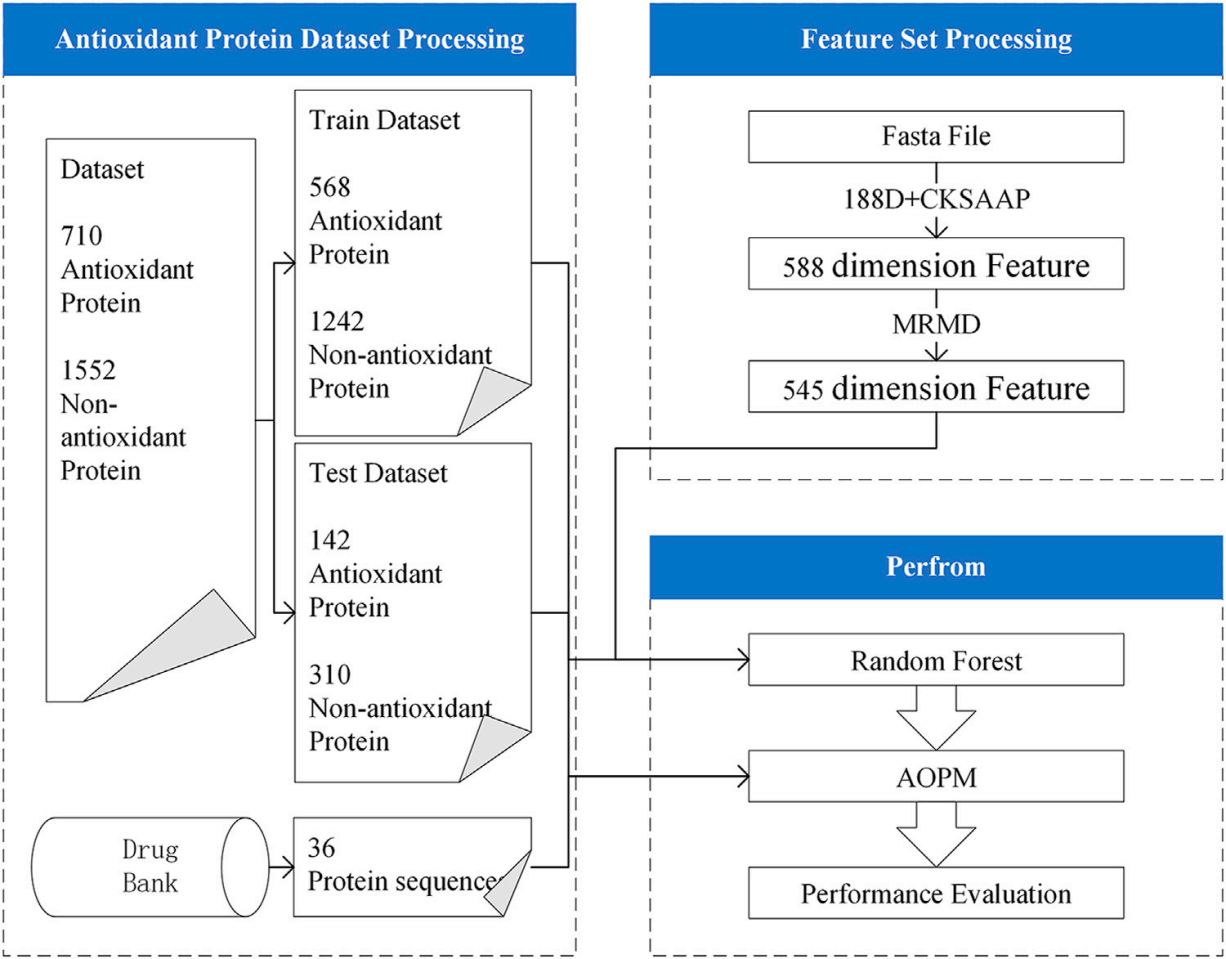
The predicted result of the enzyme of antioxidant drugs. The predicted value of protein P00441 reached 0.99, and the predicted value of protein P48506 reached 0.79. According to related literature, protein P00441 and protein P48506 are the catalytic subunits of superoxide dismutase [Cu-Zn] and glutamate-cysteine ligase, respectively. The prediction results of the remaining proteins are also around 0.6.

In DrugBank, the 36 protein sequences we screened were subjected to the same feature extraction and screening operations, and then they were input into AOPM to get their prediction results. Among them, 11 proteins were predicted to be antioxidant proteins, and the predicted value of protein P00441 reached 0.99, and the predicted value of protein P48506 reached 0.79. The predicted results are shown in Figure 5.

After consulting related literature, protein P00441 and protein P48506 were Superoxide dismutase [Cu-Zn] and Glutamate--cysteine ligase catalytic subunit, respectively. Although they play a catalytic role in antioxidants, they are also a strong antioxidant protein in themselves. We have consulted many literatures about Superoxide dismutase [Cu-Zn]. Superoxide dismutase [Cu-Zn] is the catalytic enzyme of many antioxidant drugs, and it has antioxidant properties. Although the current research does not clearly show that the remaining proteins can play an anti-oxidant effect, the sequence analysis can guide scientists to try their biological and chemical experiments.

## Conclusion

In this paper, we proposed a tool named AOPM to identify antioxidant proteins. 188D and the Composition of k-spaced Amino Acid Pairs were adopted to extract the feature set, and we selected the optional feature set with MRMD. Using the 5-fold cross-validation and random forest on the test dataset, we obtained an average accuracy of 0.920. The sensitivity, specificity, the Matthew’s Correlation Coefficient and an Area Under the Curve were 0.873, 0.942, 0.815, and 0.972, respectively. Compared with previous methods, we re-collect the antioxidant protein data. After such processing, while the proportion of positive and negative examples of the data set is reduced, the characteristics of antioxidant proteins are also strengthened, and the robustness of the trained model were greatly improved compared with existing methods. In addition, AOPM also made predictions on the real data set of DrugBank, and indeed found proteins with antioxidant properties. This work proved the feasibility of virtual drug screening based on sequence information and provided new ideas and solutions for drug development.

## Data Availability

The original contributions presented in the study are included in the article/Supplementary Material, further inquiries can be directed to the corresponding authors.

## References

[B1] Ao C. Zou Q. Yu L. (2021). RFhy-m2G: Identification of RNA N2-Methylguanosine Modification Sites Based on Random forest and Hybrid Features. Methods 21, 1046–2023. 10.1016/j.ymeth.2021.05.016 34033879

[B2] Basith S. Manavalan B. Shin T. H. Lee G. (2018). iGHBP: Computational Identification of Growth Hormone Binding Proteins from Sequences Using Extremely Randomised Tree. Comput. Struct. Biotechnol. J. 16, 412–420. 10.1016/j.csbj.2018.10.007 30425802 PMC6222285

[B3] Birben E. Sahiner U. M. Sackesen C. Erzurum S. Kalayci O. (2012). Oxidative Stress and Antioxidant Defense. World Allergy Organ. J. 5 (1), 9–19. 10.1097/WOX.0b013e3182439613 23268465 PMC3488923

[B4] Chen K. Kurgan L. Rahbari M. (2007). Prediction of Protein Crystallization Using Collocation of Amino Acid Pairs. Biochem. Biophys. Res. Commun. 355 (3), 764–769. 10.1016/j.bbrc.2007.02.040 17316561

[B5] Chen Y. Ma T. Yang X. Wang J. Song B. Zeng Y. (2021). MUFFIN: Multi-Scale Feature Fusion for Drug–Drug Interaction Prediction. Bioinformatics 37, btab169. 10.1093/bioinformatics/btab169 33720331

[B6] Cheng L. Hu Y. Sun J. Zhou M. Jiang Q. (2018). DincRNA: a Comprehensive Web-Based Bioinformatics Toolkit for Exploring Disease Associations and ncRNA Function. Bioinformatics 34 (11), 1953–1956. 10.1093/bioinformatics/bty002 29365045

[B7] Cheng L. Qi C. Zhuang H. Fu T. Zhang X. (2020). gutMDisorder: a Comprehensive Database for Dysbiosis of the Gut Microbiota in Disorders and Interventions. Nucleic Acids Res. 48 (D1), D554–D560. 10.1093/nar/gkz843 31584099 PMC6943049

[B8] Cheng L L. Qi C. Yang H. Lu M. Cai Y. Fu T. (2021). gutMGene: a Comprehensive Database for Target Genes of Gut Microbes and Microbial Metabolites. Nucleic Acids Res., gkab786. 10.1093/nar/gkab786 PMC8728193 34500458

[B9] Cheng Y Y. Gong Y. Liu Y. Song B. Zou Q. (2021). Molecular Design in Drug Discovery: a Comprehensive Review of Deep Generative Models. Brief. Bioinform. 22, bbab344. 10.1093/bib/bbab344 34415297

[B10] Ding Y. Tang J. Guo F. (2019). Protein Crystallization Identification via Fuzzy Model on Linear Neighborhood Representation. IEEE/ACM Trans. Comput. Biol. Bioinformatics. 18 (5):1986–1995. 10.1109/TCBB.2019.2954826 31751248

[B11] Dong J. Zhao M. Liu Y. Su Y. Zeng X. (2021). Deep Learning in Retrosynthesis Planning: Datasets, Models and Tools. Brief. Bioinform., bbab391. 10.1093/bib/bbab391 34571535

[B12] Dzięgielewska-Gęsiak S. Wysocka E. Michalak S. Nowakowska-Zajdel E. Kokot T. Muc-Wierzgoń M. (2014). Role of Lipid Peroxidation Products, Plasma Total Antioxidant Status, and Cu-, Zn-Superoxide Dismutase Activity as Biomarkers of Oxidative Stress in Elderly Prediabetics. Oxid. Med. Cell Longev. 2014, 987303. 10.1155/2014/987303 24891926 PMC4026982

[B13] Eleutherio E. C. A. Silva Magalhães R. S. de Araújo Brasil A. Monteiro Neto J. R. de Holanda Paranhos L. (2021). SOD1, More Than Just an Antioxidant. Arch. Biochem. Biophys. 697, 108701. 10.1016/j.abb.2020.108701 33259795

[B14] Feng P.-M. Lin H. Chen W. (2013). Identification of Antioxidants from Sequence Information Using Naive Bayes. Comput. Math. Methods Med. 2013, 567529. 10.1155/2013/567529 24062796 PMC3766563

[B15] Feng P. Chen W. Lin H. (2016). Identifying Antioxidant Proteins by Using Optimal Dipeptide Compositions. Interdiscip. Sci. Comput. Life Sci. 8 (2), 186–191. 10.1007/s12539-015-0124-9 26345449

[B16] Feng P. Ding H. Lin H. Chen W. (2017). AOD: the Antioxidant Protein Database. Sci. Rep. 7 (1), 7449–7454. 10.1038/s41598-017-08115-6 28784999 PMC5547145

[B17] Goto Y. Wysocka E. Michalak S. Nowakowska-Zajdel E. Kokot T. Muc-Wierzgoń M. (2021). Tropomyosin-related Kinase B (TrkB) Full-Length Isoform Is Related to Advanced-Stage clear Cell Ovarian Cancer (CCOC). Eur. J. Gynaecol. Oncol. 42 (5), 899–908. 10.5582/bst.8.93

[B18] Jakhar R. Dangi M. Khichi A. Chhillar A. K. (2020). Relevance of Molecular Docking Studies in Drug Designing. Curr. Bioinform. 15 (4), 270–278. 10.2174/1574893615666191219094216

[B19] Jin Q. Meng Z. Pham T. D. Chen Q. Wei L. Su R. (2019). DUNet: A Deformable Network for Retinal Vessel Segmentation. Knowledge-Based Syst. 178, 149–162. 10.1016/j.knosys.2019.04.025

[B20] Liang X. Rodríguez-Patón A. Zou Q. (2019). Molecular Computing and Bioinformatics. Molecules 24 (13), 2358. 10.3390/molecules24132358 PMC6651761 31247973

[B21] Liaw A. Wiener M. (2002). Classification and Regression by randomForest. R. News 2 (3), 18–22. 10.1021/ci034160g

[B22] Liguori I. Russo G. Curcio F. Bulli G. Aran L. Della-Morte D. (2018). Oxidative Stress, Aging, and Diseases. Clin. Interv. Aging 13, 757–772. 10.2147/CIA.S158513 29731617 PMC5927356

[B23] Liu J J. Lian X. Liu F. Yan X. Cheng C. Cheng L. (2020). Identification of Novel Key Targets and Candidate Drugs in Oral Squamous Cell Carcinoma. Curr. Bioinform. 15 (4), 328–337. 10.2174/1574893614666191127101836

[B24] Liu T T. Chen J. M. Zhang D. Zhang Q. Peng B. Xu L. (2020). ApoPred: Identification of Apolipoproteins and Their Subfamilies with Multifarious Features. Front. Cel Dev. Biol. 8, 621144. 10.3389/fcell.2020.00234 PMC7820372 33490085

[B25] Long H. Sun Z. Li M. Fu H. Y. Lin M. C. (2020). Predicting Protein Phosphorylation Sites Based on Deep Learning. Curr. Bioinform. 15 (4), 300–308. 10.2174/1574893614666190902154332

[B26] Long J. Yang H. Yang Z. Jia Q. Liu L. Kong L. (2021). Integrated Biomarker Profiling of the Metabolome Associated with Impaired Fasting Glucose and Type 2 Diabetes Mellitus in Large-Scale Chinese Patients. Clin. Transl. Med. 11 (6), e432. 10.1002/ctm2.432 34185410 PMC8167862

[B27] Lv Z. Jin S. Ding H. Zou Q. (2019). A Random Forest Sub-golgi Protein Classifier Optimized via Dipeptide and Amino Acid Composition Features. Front. Bioeng. Biotechnol. 7, 215. 10.3389/fbioe.2019.00215 31552241 PMC6737778

[B28] Lv Z. Wang P. Zou Q. Jiang Q. (2020). Identification of Sub-golgi Protein Localization by Use of Deep Representation Learning Features. Bioinformatics 36 (24), 5600–5609. 10.1093/bioinformatics/btaa1074 PMC8023683 33367627

[B29] Lv Z. Zhang J. Ding H. Zou Q. (2020). RF-PseU: A Random Forest Predictor for RNA Pseudouridine Sites. Front. Bioeng. Biotechnol. 8, 134. 10.3389/fbioe.2020.00134 32175316 PMC7054385

[B30] Lv Z. Wang D. Ding H. Zhong B. Xu L. (2020). Escherichia Coli DNA N-4-Methycytosine Site Prediction Accuracy Improved by Light Gradient Boosting Machine Feature Selection Technology. IEEE Access 8, 14851–14859. 10.1109/access.2020.2966576

[B31] Lv Z Z. Cui F. Zou Q. Zhang L. Xu L. (2021). Anticancer Peptides Prediction with Deep Representation Learning Features. Brief. Bioinform. 22, bbab008. 10.1093/bib/bbab008 33529337

[B32] Lv H H. Dao F. Y. Zulfiqar H. Lin H. (2021). DeepIPs: Comprehensive Assessment and Computational Identification of Phosphorylation Sites of SARS-CoV-2 Infection Using a Deep Learning-Based Approach. Brief. Bioinform. 22 (6), 244. 10.1093/bib/bbab244 PMC8406875 34184738

[B33] Lyu Y. He W. Li S. Zou Q. Guo F. (2020). iPro2L-PSTKNC: a Two-Layer Predictor for Discovering Various Types of Promoters by Position Specific of Nucleotide Composition. IEEE J. Biomed. Health Inform. 25 (6), 2329–2337. 10.1109/JBHI.2020.3026735 32976109

[B34] Manavalan B. Basith S. Shin T. H. Wei L. Lee G. (2019a). Meta-4mCpred: A Sequence-Based Meta-Predictor for Accurate DNA 4mC Site Prediction Using Effective Feature Representation. Mol. Ther. Nucleic Acids 16, 733–744. 10.1016/j.omtn.2019.04.019 31146255 PMC6540332

[B35] Manavalan B. Basith S. Shin T. H. Wei T. Lee G. (2019b). mAHTPred: a Sequence-Based Meta-Predictor for Improving the Prediction of Anti-hypertensive Peptides Using Effective Feature Representation. Bioinformatics 35 (16), 2757–2765. 10.1093/bioinformatics/bty1047 30590410

[B36] Meng C. Jin S. Wang L. Guo F. Zou Q. (2019). AOPs-SVM: a Sequence-Based Classifier of Antioxidant Proteins Using a Support Vector Machine. Front. Bioeng. Biotechnol. 7, 224. 10.3389/fbioe.2019.00224 31620433 PMC6759716

[B37] Mo F. Luo Y. Fan D. Zeng H. Zhao Y. Luo M. (2020). Integrated Analysis of mRNA-Seq and miRNA-Seq to Identify C-MYC, YAP1 and miR-3960 as Major Players in the Anticancer Effects of Caffeic Acid Phenethyl Ester in Human Small Cell Lung Cancer Cell Line. Curr. Gene Ther. 20 (1), 15–24. 10.2174/1566523220666200523165159 32445454

[B38] Naseer S. Hussain W. Khan Y. D. Rasool N. (2020). Sequence-based Identification of Arginine Amidation Sites in Proteins Using Deep Representations of Proteins and PseAAC. Curr. Bioinformatics 15 (8), 937–948. 10.2174/1574893615666200129110450.

[B39] Niu K. Luo X. Zhang S. Teng Z. Zhang T. Zhao Y. (2021). iEnhancer-EBLSTM: Identifying Enhancers and Strengths by Ensembles of Bidirectional Long Short-Term Memory. Front. Genet. 12, 385. 10.3389/fgene.2021.665498 PMC8021722 33833783

[B40] Raghunath A. Sundarraj K. Nagarajan R. Arfuso F. Bian J. Kumar A. P. (2018). Antioxidant Response Elements: Discovery, Classes, Regulation and Potential Applications. Redox Biol. 17, 297–314. 10.1016/j.redox.2018.05.002 29775961 PMC6007815

[B41] Riaz F. Li D. (2019). Non-coding RNA Associated Competitive Endogenous RNA Regulatory Network: Novel Therapeutic Approach in Liver Fibrosis. Curr. Gene Ther. 19 (5), 305–317. 10.2174/1566523219666191107113046 31696817

[B42] Ściskalska M. Ołdakowska M. Marek G. Milnerowicz H. (2020). Changes in the Activity and Concentration of Superoxide Dismutase Isoenzymes (Cu/Zn SOD, MnSOD) in the Blood of Healthy Subjects and Patients with Acute Pancreatitis. Antioxidants 9 (10), 948. 10.3390/antiox9100948 PMC7601220 33019780

[B43] Shaker B. Tran K. M. Jung C. Na D. (2021). Introduction of Advanced Methods for Structure-Based Drug Discovery. Curr. Bioinform. 16 (3), 351–363. 10.2174/1574893615999200703113200

[B44] Sheng Y. Jiang Y. Yang Y. Li X. Qiu J. Wu J. (2021). CNA2Subpathway: Identification of Dysregulated Subpathway Driven by Copy Number Alterations in Cancer. Brief. Bioinform. 22 (5), bbaa413. 10.1093/bib/bbaa413 33423051

[B45] Su R. Liu X. Wei L. Zou Q. (2019). Deep-Resp-Forest: A Deep forest Model to Predict Anti-cancer Drug Response. Methods 166, 91–102. 10.1016/j.ymeth.2019.02.009 30772464

[B46] Tiwari M. K. Hägglund P. M. Møller I. M. Davies M. J. Bjerrum M. J. (2019). Copper Ion / H2O2 Oxidation of Cu/Zn-Superoxide Dismutase: Implications for Enzymatic Activity and Antioxidant Action. Redox Biol. 26, 101262. 10.1016/j.redox.2019.101262 31284117 PMC6614508

[B47] Triguero I. González S. Moyano J. M. García S. Alcalá-Fdez J. Luengo J. (2017). KEEL 3.0: an Open Source Software for Multi-Stage Analysis in Data Mining. Int. J. Comput. Int. Sys. 10 (1), 1238–1249. 10.2991/ijcis.10.1.82

[B48] Usman M. Lee J. A. (2019). “Afp-cksaap: Prediction of Antifreeze Proteins Using Composition of K-Spaced Amino Acid Pairs with Deep Neural Network,” in IEEE 19th International Conference on Bioinformatics and Bioengineering (BIBE), Athens, Greece, 26 December, 2019 (Athens, Greece: IEEE). 10.1109/bibe.2019.00016

[B49] Wang G. Wang Y. Feng W. Wang X. Yang J. Y. Zhao Y. (2008). Transcription Factor and microRNA Regulation in Androgen-dependent and -independent Prostate Cancer Cells. BMC Genomics 9 (2), S22–S12. 10.1186/1471-2164-9-S2-S22 PMC2559887 18831788

[B50] Wang G. Wang Y. Teng M. Zhang D. Li L. Liu Y. (2010). Signal Transducers and Activators of Transcription-1 (STAT1) Regulates microRNA Transcription in Interferon Gamma-Stimulated HeLa Cells. PLoS One 5 (7), e11794. 10.1371/journal.pone.0011794 20668688 PMC2909916

[B51] Wang G. Luo X. Wang J. Wan J. Xia S. Zhu H. (2018). MeDReaders: a Database for Transcription Factors that Bind to Methylated DNA. Nucleic Acids Res. 46 (D1), D146–D151. 10.1093/nar/gkx1096 29145608 PMC5753207

[B52] Wang Y. Ding Y. Tang Y. Dai Y. Guo F. (2019). CrystalM: a Multi-View Fusion Approach for Protein Crystallization Prediction. IEEE/ACM Trans. Comput. Biol. Bioinformatics 18, 325. 10.1109/tcbb.2019.2912173 31027046

[B53] Wang X.-F. Gao P. Liu Y.-F. Li H.-F. Lu F. (2020). Predicting Thermophilic Proteins by Machine Learning. Curr. Bioinform. 15 (5), 493–502. 10.2174/1574893615666200207094357

[B54] Wei L. Liao M. Gao Y. Ji R. He Z. Zou Q. (2014). Improved and Promising Identification of Human MicroRNAs by Incorporating a High-Quality Negative Set. IEEE/ACM Trans. Comput. Biol. Bioinform. 11 (1), 192–201. 10.1109/TCBB.2013.146 26355518

[B55] Wei L. Xing P. Zeng J. Chen J. Su R. Guo F. (2017). Improved Prediction of Protein-Protein Interactions Using Novel Negative Samples, Features, and an Ensemble Classifier. Artif. Intell. Med. 83, 67–74. 10.1016/j.artmed.2017.03.001 28320624

[B56] Wei L. Zhou C. Chen H. Song J. Su R. (2018). ACPred-FL: a Sequence-Based Predictor Using Effective Feature Representation to Improve the Prediction of Anti-cancer Peptides. Bioinformatics 34 (23), 4007–4016. 10.1093/bioinformatics/bty451 29868903 PMC6247924

[B57] Wishart D. S. Feunang Y. D. Guo A. C. Lo E. J. Marcu A. Grant J. R. (2018). DrugBank 5.0: a Major Update to the DrugBank Database for 2018. Nucleic Acids Res. 46 (D1), D1074–D1082. 10.1093/nar/gkx1037 29126136 PMC5753335

[B58] Wu X. Yu L. (2021). EPSOL: Sequence-Based Protein Solubility Prediction Using Multidimensional Embedding. Bioinformatics 37, 4314. 10.1093/bioinformatics/btab463 34145885

[B59] Wu D. Lv Z. Xu X. Yin Z. Lou H. (2021). Clinicopathological Features and Prognostic Factors for Survival and Lymph Node Metastases in Stage IB Adenocarcinoma of the Cervix. Eur. J. Gynaecol. Oncol. 42 (3), 450–456. 10.31083/j.ejgo.2021.03.2300

[B60] Xu L. Liang G. Shi S. Liao C. (2018). SeqSVM: a Sequence-Based Support Vector Machine Method for Identifying Antioxidant Proteins. Int. J. Mol. Sci. 19 (6), 1773. 10.3390/ijms19061773 PMC6032279 29914044

[B61] Yan N. Lv Z. Hong W. Xu X. (2021). Editorial: Feature Representation and Learning Methods with Applications in Protein Secondary Structure. Front. Bioeng. Biotechnol. 9 (822), 748722. 10.3389/fbioe.2021.748722 34568304 PMC8458893

[B62] Yang Y. Fan C. Zhao Q. (2020). Recent Advances on the Machine Learning Methods in Identifying Phage Virion Proteins. Curr. Bioinform. 15 (7), 657–661. 10.2174/1574893614666191203155511

[B63] Yang H. Luo Y. Ren X. Wu M. He X. Peng B. (2021). Risk Prediction of Diabetes: Big Data Mining with Fusion of Multifarious Physical Examination Indicators. Inf. Fusion 75, 140–149. 10.1016/j.inffus.2021.02.015

[B64] Yu XP X.-P. Zhang Z. Pu L Tang T Guo F., (2021). Breast Cancer Overall-Survival Can Be Predicted with a 19 lncRNA Tissue Signature. Eur. J. Gynaecol. Oncol. 42 (5), 838–843. 10.31083/j.ejgo4205128

[B65] Yu L L. Xia M. An Q. (2021). A Network Embedding Framework Based on Integrating Multiplex Network for Drug Combination Prediction. Brief. Bioinform., bbab364. 10.1093/bib/bbab364 34505623

[B66] Zeng X. Song X. Ma T. Pan X. Zhou Y. Hou Y. (2020). Repurpose Open Data to Discover Therapeutics for COVID-19 Using Deep Learning. J. Proteome Res. 19 (11), 4624–4636. 10.1021/acs.jproteome.0c00316 32654489

[B67] Zeng X. Zhu S. Hou Y. Zhang P. Li L. Li J. (2020). Network-based Prediction of Drug-Target Interactions Using an Arbitrary-Order Proximity Embedded Deep forest. Bioinformatics 36 (9), 2805–2812. 10.1093/bioinformatics/btaa010 31971579 PMC7203727

[B68] Zeng X. Tu X. Liu Y. Fu X. Su Y. (2022). Toward Better Drug Discovery with Knowledge Graph. Curr. Opin. Struct. Biol. 72, 114–126. 10.1016/j.sbi.2021.09.003 34649044

[B69] Zhai Y. Chen Y. Teng Z. Zhao Y. (2020). Identifying Antioxidant Proteins by Using Amino Acid Composition and Protein-Protein Interactions. Front Cel Dev. Biol. 8, 591487. 10.3389/fcell.2020.591487 PMC7658297 33195258

[B70] Zhang CH C. H. Li M. Lin Y. P. Gao Q. (2020). Systemic Therapy for Hepatocellular Carcinoma: Advances and Hopes. Curr. Gene Ther. 20 (2), 84–99. 10.2174/1566523220666200628014530 32600231

[B71] Zhang J J. Zhang Z. Pu L. Tang T. Guo F. (2020). AIEpred: an Ensemble Predictive Model of Classifier Chain to Identify Anti-inflammatory Peptides. IEEE/ACM Trans. Comput. Biol. Bioinformatics. 18 (5):1831–1840. 10.1109/TCBB.2020.2968419 31985437

[B72] Zhang D. Chen H. D. Zulfiqar H. Yuan S. S. Huang Q. L. Zhang Z. Y. (2021). iBLP: An XGBoost-Based Predictor for Identifying Bioluminescent Proteins. Comput. Math. Methods Med. 2021, 6664362. 10.1155/2021/6664362 33505515 PMC7808816

[B73] Zhao S S. Ju Y. Ye X. Zhang J. Han S. (2021). Bioluminescent Proteins Prediction with Voting Strategy. Curr. Bioinform. 16 (2), 240–251. 10.2174/1574893615999200601122328

[B74] Zhao X X. Lv Z. Xu X. Yin Z. Lou H., (2021). Identifying Plant Pentatricopeptide Repeat Proteins Using a Variable Selection Method. Front. Plant Sci. 12, 298. 10.3389/fpls.2021.506681 PMC7957076 33732270

[B75] Zhu L. Duan G. Yan C. Wang J. (2021). Prediction of Microbe-Drug Associations Based on Chemical Structures and the KATZ Measure. Curr. Bioinform. 16 (6), 807–819. 10.2174/1574893616666210204144721

[B76] Zia A. Farkhondeh T. Pourbagher-Shahri A. M. Samarghandian S. (2021). The Role of Curcumin in Aging and Senescence: Molecular Mechanisms. Biomed. Pharmacother. 134, 111119. 10.1016/j.biopha.2020.111119 33360051

[B77] Zou Q. Zeng J. Cao L. Ji R. (2016). A Novel Features Ranking Metric with Application to Scalable Visual and Bioinformatics Data Classification. Neurocomputing 173, 346–354. 10.1016/j.neucom.2014.12.123

[B78] Zulfiqar H. Yuan S.-S. Huang Q.-L. Sun Z.-J. Dao F.-Y. Yu X.-L. (2021). Identification of Cyclin Protein Using Gradient Boost Decision Tree Algorithm. Comput. Struct. Biotechnol. J. 19, 4123–4131. 10.1016/j.csbj.2021.07.013 34527186 PMC8346528

[B79] Zulfiqar H. Khan R. S. Hassan F. Hippe K. Hunt C. Ding H. (2021). Computational Identification of N4-Methylcytosine Sites in the Mouse Genome with Machine-Learning Method. Math. Biosci. Eng. 18 (4), 3348–3363. 10.3934/mbe.2021167 34198389

